# Identification and Validation of a Novel Tumor Microenvironment-Related Prognostic Signature of Patients With Hepatocellular Carcinoma

**DOI:** 10.3389/fmolb.2022.917839

**Published:** 2022-06-30

**Authors:** Rui Li, Weiheng Zhao, Rui Liang, Chen Jin, Huihua Xiong

**Affiliations:** ^1^ Department of Oncology, Tongji Hospital, Huazhong University of Science and Technology, Wuhan, China; ^2^ Biological Engineering Academy, Chongqing University, Chongqing, China; ^3^ Department of Epidemiology and Biostatistics, School of Public Health and Management, Wenzhou Medical University, Wenzhou, China

**Keywords:** hepatocellular carcinoma, tumor microenvironment, WGCNA, ESTIMATE score, immune score

## Abstract

**Background:** In recent years, immunotherapy has changed the therapeutic landscape of hepatocellular carcinoma (HCC). Since the efficacy of immunotherapy is closely related to the tumor microenvironment (TME), in this study, we constructed a prognostic model based on TME to predict the prognosis and immunotherapy effect of HCC patients.

**Methods:** Transcriptome and follow-up data of 374 HCC patients were acquired from the TCGA Cancer Genome Atlas (TCGA) database. The immune/stromal/estimate scores (TME scores) and tumor purity were calculated using the ESTIMATE algorithm and the module most associated with TME scores were screened by the weighted gene co-expression network analysis (WGCNA). A TME score-related prognostic model was constructed and patients were divided into a high-risk group and a low-risk group. Kaplan-Meier survival curves and receiver operator characteristic curve (ROC) were used to evaluate the performance of the TME risk prognostic model and validated with the external database International Cancer Genome Consortium (ICGC) cohort. Combined with clinicopathologic factors, a prognostic nomogram was established. The nomogram’s ability to predict prognosis was assessed by ROC, calibration curve, and the decision curve analysis (DCA). Gene Set Enrichment Analyses (GSEA) were conducted to explore the underlying biological functions and pathways of this risk signature. Moreover, the possible correlation of risk signature with TME immune cell infiltration, immune checkpoint inhibitor (ICI) treatment response, single-nucleotide polymorphisms (SNPs), and drug sensitivity were assessed. Finally, real-time PCR was used to verify the gene expression levels in normal liver cells and cancer cells.

**Results:** KM survival analysis results indicated that high immune/stromal/estimate score groups were closely associated with a better prognosis, while the tumor purity showed a reverse trend (*p* < 0.01). WGCNA demonstrated that the yellow module was significantly correlated with the TME score. The 5-genes TME risk signature was built to predict the prognosis of patients with HCC including *DAB2*, *IL18RAP*, *RAMP*3, *FCER1G*, and *LHFPL2*. Patients with a low-risk score have higher levels of tumor-infiltrating immune cells and higher expression of immune checkpoints, which may be more sensitive to immunotherapy.

**Conclusion:** It provided a theoretical basis for predicting the prognosis and personalized treatment of patients with HCC.

## Introduction

Hepatocellular carcinoma (HCC) is ranked the sixth most common malignant tumor and is one of the causes of cancer-related deaths (Bray et al., 2018). Therefore, it is a major public health challenge. The recognized risk factors include chronic infection with hepatitis virus, excessive alcohol intake, smoking, and metabolic syndrome such as nonalcoholic fatty liver disease (NAFLD), type II diabetes, and abdominal obesity (Llovet et al., 2021; McGlynn et al., 2021). Early-stage patients can be treated with radical surgery or liver transplantation (Gunasekaran et al., 2021), but due to the complex internal tissue structure and insidious onset of HCC, most patients are already advanced at the time of diagnosis, resulting in a poor prognosis with an overall 5-year survival (OS) rate of less than 10% (Forner et al., 2018). Systemic chemotherapy and targeted therapy have limited efficacy in advanced HCC (Chen et al., 2019). For example, as the first-line treatment of choice for patients with advanced HCC, sorafenib markedly extended median survival but long-term use resulted in additional toxic side effects and progression of drug resistance in subsequent treatment (Abou-Alfa et al., 2006; Cheng et al., 2020). Therefore, there is still a need for a breakthrough in treatment. In recent years, immunotherapy (mainly in the form of immune checkpoint inhibitors—ICIs) has changed the landscape of cancer treatment, particularly in melanoma (Brower, 2015; Buchbinder and Hodi, 2016), and is currently being conducted on a broad range of neoplasms, including HCC (Llovet et al., 2018; Sangro et al., 2021). The available immune checkpoint inhibitors are antibodies that activate T-cell-mediated antitumor responses by selectively blocking checkpoint receptors PD-1, PD-L1, and CTLA-4. Among them, nivolumab, pembrolizumab (PD-1 inhibitor), and tremelimumab (CTLA-4 inhibitor) have been shown to be safe and effective in clinical trials; therefore, pembrolizumab (Zhu et al., 2018; Finn et al., 2020) and nivolumab (El-Khoueiry et al., 2017; Yau et al., 2019) have been approved by the U.S. Food and Drug Administration as the second-line treatment for HCC. However, ICI therapy has a lasting effect in only a subset of patients, and most of the patients do not respond to ICI monotherapy (Qin et al., 2019). Emerging evidence indicates that tumor microenvironment (TME) is not only related to tumorigenesis, progression, and prognosis but also closely involved in the efficacy of immunotherapy (Hinshaw and Shevde, 2019; Bader et al., 2020).

TME consists of a heterogeneous population of cancer cells themselves, infiltrating immune cells, stromal cells, endothelial cells, cancer-associated fibroblasts, extracellular matrix molecules, and inflammatory mediators (cytokines, chemokines) (Anderson and Simon, 2020). Among them, immune cells and stromal cells are the main components of the tumor microenvironment that perform various biological functions (Yin et al., 2018; Hao et al., 2021). For example, in yupei Chen’s study (Chen et al., 2020), the single-cell sequencing analysis showed that macrophages, CLEC9A^+^ DCs, natural killer cells (NK), and plasma cells were significantly correlated with better survival outcomes in patients with nasopharyngeal carcinoma. In multiple tumors, high infiltration of monocyte-derived macrophages (Mφ) is associated with poor prognosis, and Mφ can release factors such as EGF to promote cancer cell motility and invasion. Except for immune cells, stromal cells—endothelial cells can promote tumor tolerance by regulating antigen presentation and homing activity of immune cells (Savinov et al., 2003). Therefore, exploring the signature of immune and stromal cell-related genes in the tumor microenvironment could offer new insights into HCC and forecast prognosis and ICI treatment response. In 2013, the ESTIMATE algorithm was developed by Yoshihara et al. (2013) to deduce the proportion of stromal cells and immune cells in malignant tumors through gene expression profiles. In the last few years, the ESTIMATE algorithm has been introduced to lung cancer (Wu et al., 2021), esophageal cancer (Qu et al., 2021), breast cancer (Xu et al., 2021a), cervical cancer (Yu et al., 2021), and so on. This bioinformatics analysis provides a powerful tool for evaluating the TME status in malignant tumors. In this study, based on the gene expression data from TCGA and ICGC databases, we applied the estimate algorithm and WGCNA (Langfelder et al., 2008) methods to construct a TME score-related prognostic model to predict the prognosis of HCC patients and the efficacy of immunotherapy.

## Materials and Methods

### Data Sources

RNAseq mRNA and clinical data of patients with HCC were achieved from the TCGA (https://portal.gdc.cancer.gov/) and ICGC (https://dcc.icgc.org/) databases. To ensure the different databases were comparable, we applied the scale method to normalize the gene expression data. Patients with pathologically confirmed HCC and survival time greater than 30 days should be considered. Finally, a total of 602 HCC cases were included in TCGA and ICGA databases (TCGA: 342, ICGA: 260) for further analysis.

### TME Score and Correlation of Clinical Parameters

Based on gene expression profiles, the ESTIMATE algorithm was utilized to calculate the infiltration levels of stromal/immune cells. The relationship between immune/stromal/estimate score (TME score) and tumor purity was evaluated by the spearman correlation test. According to clinical parameters, the Wilcox test was applied to compare the relationship between TME score and subgroups. Then, we determined the optimal cut-off value of the TME score based on the log-rank test and the surv cutpoint () function and divided the patients into high and low scoring groups according to the cut-off value, and finally plotted the K-M survival curves.

### Gene Co-Expression Network Analysis and Identification of TME-Related Modules

WGCNA is a systematic biological approach developed by Langfelder et al. (2008), aiming at finding co-expressed gene modules and exploring the relationship between gene networks and the phenotypes of interest as well as the core genes in the networks. The WGCNA package in R was used for gene module clustering and visualization.

### Screen the Differentially Expressed Genes

Differential genes in the TME score and tumor purity in high/low groups were screened by limma package (FDR <0.05, |logFC| >1), and ggplot2 was used to map the volcano plot. The Venn diagram package intersects the screened differential genes with the yellow modules in WGCNA. The intersected genes of immune/stromal score and WGCNA were obtained for subsequent analysis.

### Development and Validation of the TME-Related Prognostic Signature for HCC

Lasso regression analysis was performed for dimensionality reduction of the intersected genes. Univariate cox regression analysis identified 9 genes associated with HCC prognosis. Then, the TME score-related prognostic risk signature was optimized by forward and backward inclusion and the minimum AIC value was obtained according to different fitting results. Finally, five gene construction models were obtained: *DAB2*, *IL18RAP*, *RAMP3*, *FCER1G*, and *LHFPL2*. Risk score = (0.257 × *DAB2*) + (−0.607 × *IL18RAP*) + (−0.314 × *RAMP3*) + (0.168 × *FCER1G*) + (0.327 × *LHFPL2*). The risk score for each patient was calculated according to this formula. The OS predictive performance of the prognostic risk model was evaluated between the training (TCGA) and validation cohorts (ICGC) by dividing patients into low- and high-risk groups based on the median and optimal cut-off point. In addition, the AUC values further confirm the predictive sensitivity and specificity of the TME risk signature. A nomogram combining risk scores and clinicopathological parameters predicting prognosis in HCC was constructed with the “rms” R package. The discernment of our nomogram was assessed by ROC curves. The calibration curve was plotted to investigate the conformity between the actual OS and the OS predicted by the nomogram. Decision curve analysis (DCA) was plotted to estimate the clinical application value.

### Gene Set Enrichment Analyses

To better access the biological functions and pathways of the TME score risk signature, we performed Gene Set Enrichment Analyses (GSEA) through R packages “clusterProfiler,” “enrichplot,” and “ggplot2.” The gene sets “c5.go.v7.4. symbols.gmt,” “c2.cp.kegg.v7.4. symbols.gmt,” and “h.all.v7.4.symbols.gmt” were chosen as the reference gene set. The normalized enrichment score (|NES| >1), nominal *p* value < 0.05 (NOM *p* value), and FDR adjusted *q*-value < 0.25 were considered as significant pathway enrichment.

### Risk Scores Correlated With Tumor Microenvironment, ICI Treatment Response, Single-Nucleotide Polymorphisms, and Drug Sensitivity

To explore the immune cell infiltration in HCC patients with high- and low-risk groups, based on the R package “immunedeconv,” we adopted seven common suitable methods, including MCPCOUNTER, XCELL, TIMER, QUANTISEQ, CIBERSORT-ABS, EPIC, and CIBERSORT. CIBERSORT was also applied to calculate the proportion of 22 types of immune cells in each HCC patient. Tumor immune dysfunction and exclusion (TIDE) score integrates the characteristics of T-cell dysfunction and removal and simulates tumor immune escape with different levels of tumor-infiltrating cytotoxic T cells. Compared with other biomarkers, the TIDE score has prominent advantages. Therefore, we calculated the scores of TIDE, Dysfunction, Exclusion, and MSI in each patient (http://tide.dfci.harvard.edu/). Single-nucleotide polymorphism (SNP) analysis was conducted by the R package maftools to explore the gene mutation profile of the risk signature. IC50 was calculated utilizing the R package pRRophetic, and the Wilcoxon test was used to assess the IC50 for the high- and low-risk groups. The spearman correlation analysis was used to analyze the correlation between risk score and immune cells, immune checkpoints, and drug sensitivity.

### Statistical Analysis

R (version 4.0.3) and the associated packages were used for all computational and statistical studies. Two-tailed *p* values < 0.05 were considered statistically significant.

## Results

### Association Between TME-Related Risk Score and Clinicopathological Features of HCC Patients

**FIGURE 1:**
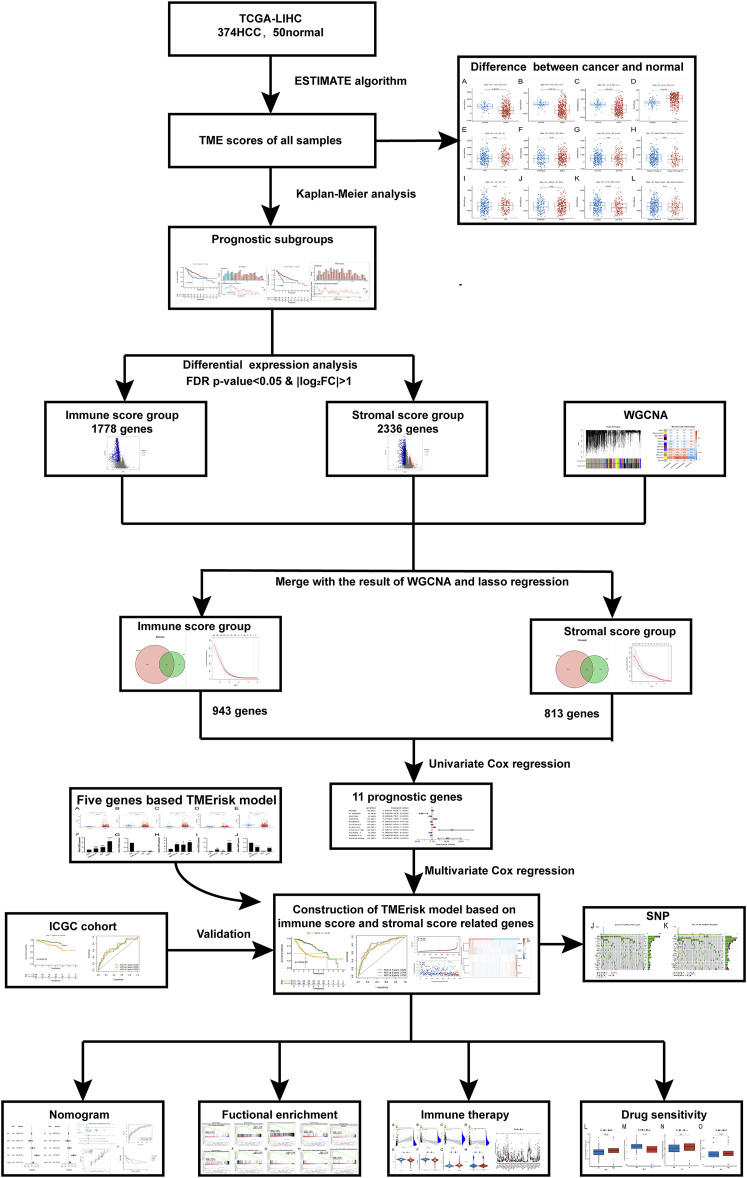
The brief flowchart of this study.

**FIGURE 2:**
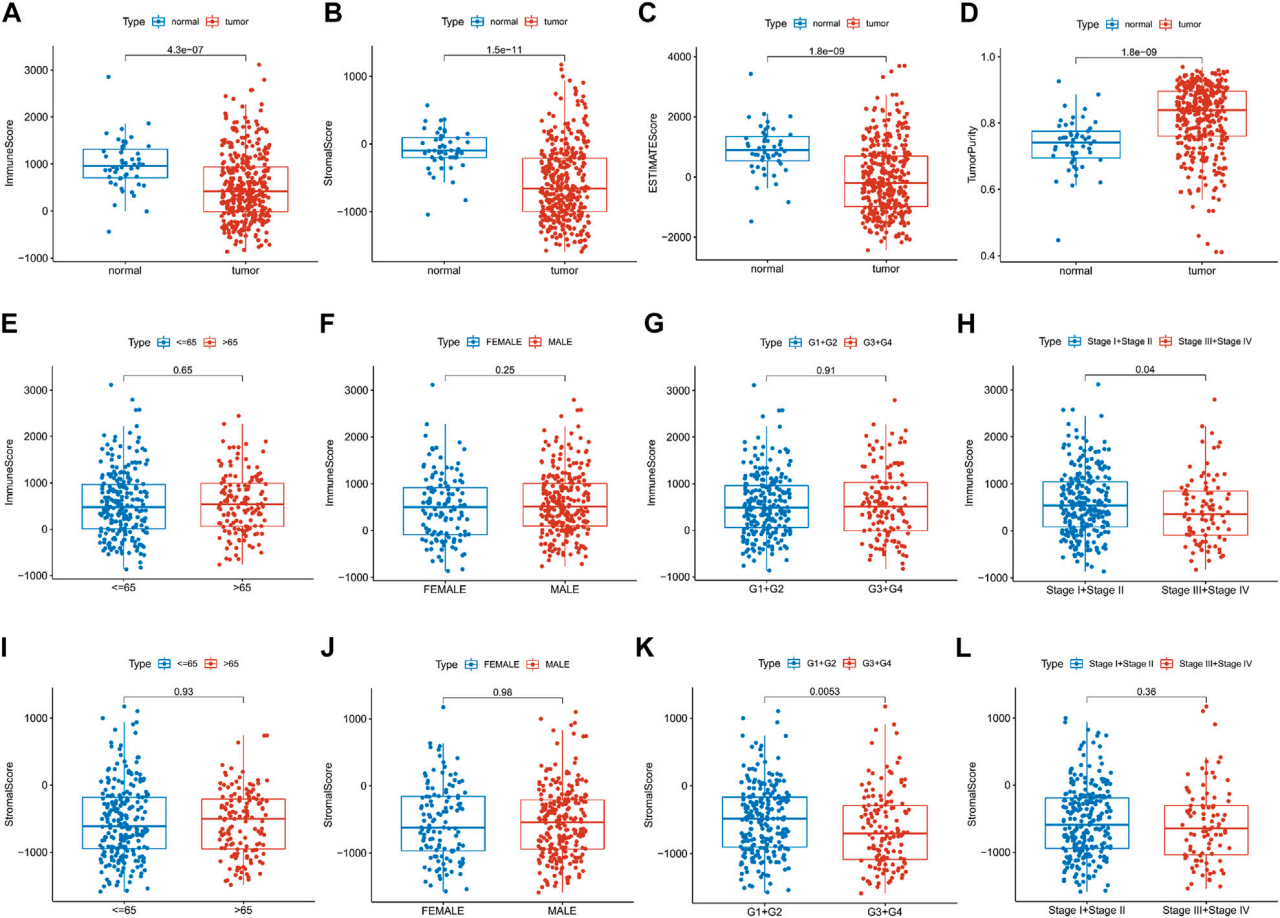
Relationship between the TME score and clinical features. **(A–D)** Differential expression of the TME score and tumor purity in the HCC patients’ normal and cancer tissue. **(E–L)** Immune/stromal score distribution differences in age, sex, grade and stage subgroups.

**FIGURE 3:**
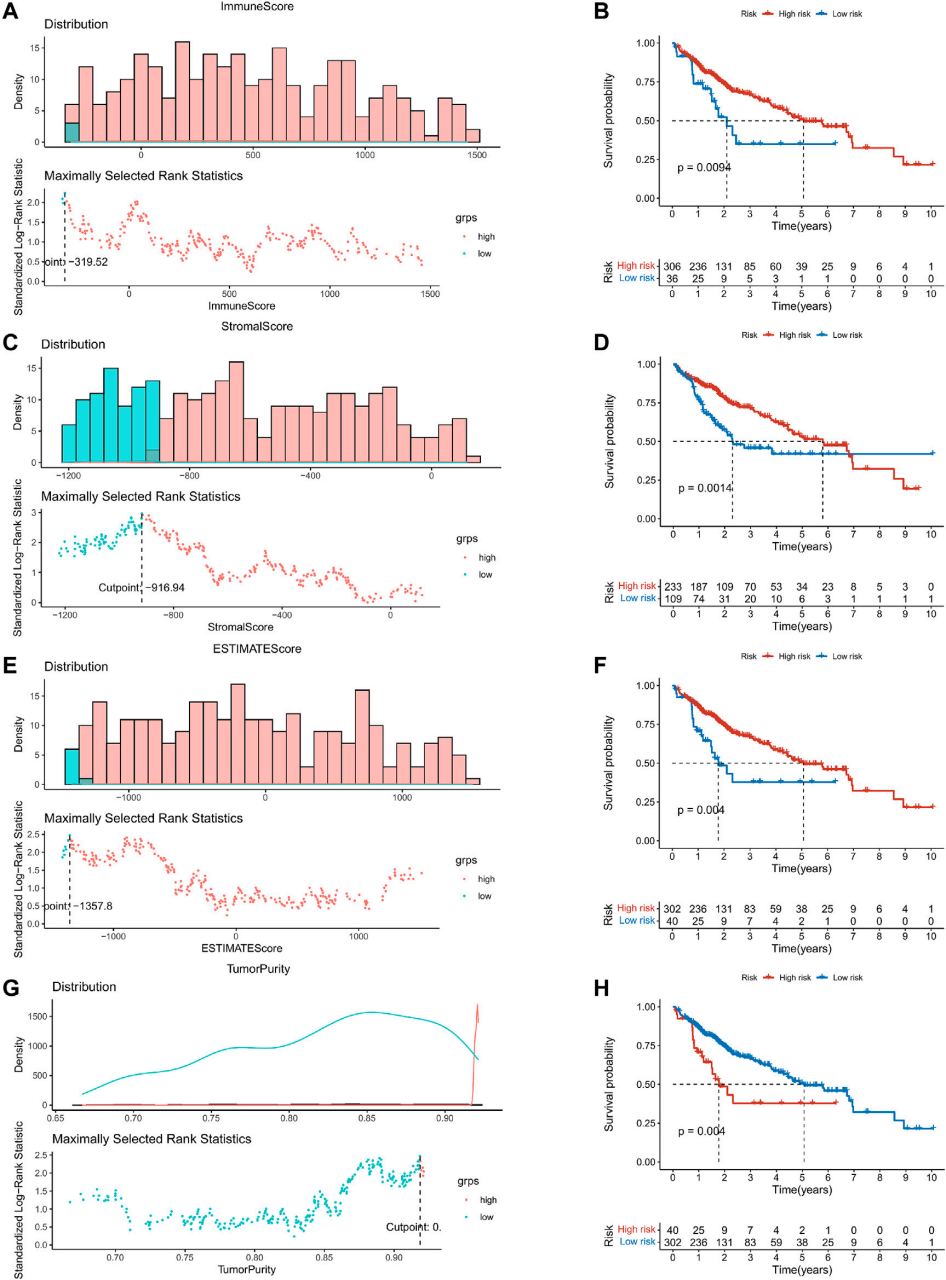
K–M survival analysis based on the optimal cut-off value of the TME score. **(A,C,E,G)** The optimal cut-off value of the TME score and tumor purity were determined and visualized. **(B,D,F,H)** The Kaplan–Meier survival analysis in the high- and low-risk groups.

This study was carried out following the flowchart shown in Figure 1. The RNAseq data of 374 cancer tissues and 50 para-cancer tissues were obtained from the TCGA database. After eliminating patients with a survival of 30 days or less, 342 patients remained (TCGA cohort). Meanwhile, gene expression and clinical data of 260 HCC patients were downloaded from the ICGC database (LIRI-JP cohort). The clinical baseline of patients with HCC was shown in Table 1. First, we calculated each patient’s immune/stromal/estimate score (TME score) and tumor purity based on gene expression profile by the ESTIMATE algorithm. It was observed that tumor purity was higher in tumor tissues, while the TME score were higher in normal tissues (Figures 2A–D). In addition, the TME score was negatively related to the tumor purity (Supplementary Figure S1). Then, the correlation between the TME score and clinicopathological features was explored (Figures 2E–L). Patients with high immune scores had a better clinical stage (*p* = 0.04, Figure 2H). In the stromal score group, grade G3/G4 (poorly differentiated) had a significantly lower stromal score than G1/G2 (*p* < 0.0053, Figure 2K). Finally, the optimal cut-off value of the TME score was determined and visualized (Figures 3A,C,E,G). The K-M survival analysis showed that the higher the TME score group had a good prognosis and the lower the purity of the tumor, the better the prognosis (Figures 3B,D,F,H).

**TABLE 1:** Summary of HCC patient clinical parameters in the TCGA and LIRI-JP cohort.

	TCGA-cohort	LIRI-JP cohort
Number of patients	342	260
Age		
≤65	216 (63.2%)	98 (37.7%)
>65	126 (36.8%)	162 (62.3%)
Gender		
Female	109 (31.9%)	68 (26.2%)
Male	233 (68.1%)	192 (73.8%)
Grade		
G1	53 (15.5%)	NA
G2	161 (47.1%)	NA
G3	111 (32.5%)	NA
G4	12 (3.5%)	NA
Unknown	5 (1.5%)	NA
Stage		
Stage I	161 (47.1%)	40 (15.4%)
Stage II	77 (22.5%)	117 (45.0%)
Stage III	80 (23.4%)	80 (30.8%)
Stage IV	3 (0.9%)	23 (8.8%)
Unknown	21 (6.1%)	NA
T-stage		
T1	168 (49.1%)	NA
T2	84 (24.6%)	NA
T3	74 (21.6%)	NA
T4	13 (3.8%)	NA
TX	1 (0.3%)	NA
Unknown	2 (0.6%)	NA
N-stage		
N0	239 (69.9%)	NA
N1	3 (0.9%)	NA
NX	99 (28.9%)	NA
Unknown	1 (0.3%)	NA
M-stage		
M0	244 (71.3%)	NA
M1	3 (0.9%)	NA
MX	95 (27.8%)	NA

### Weighted Gene Co-Expression Network Analysis

**FIGURE 4:**
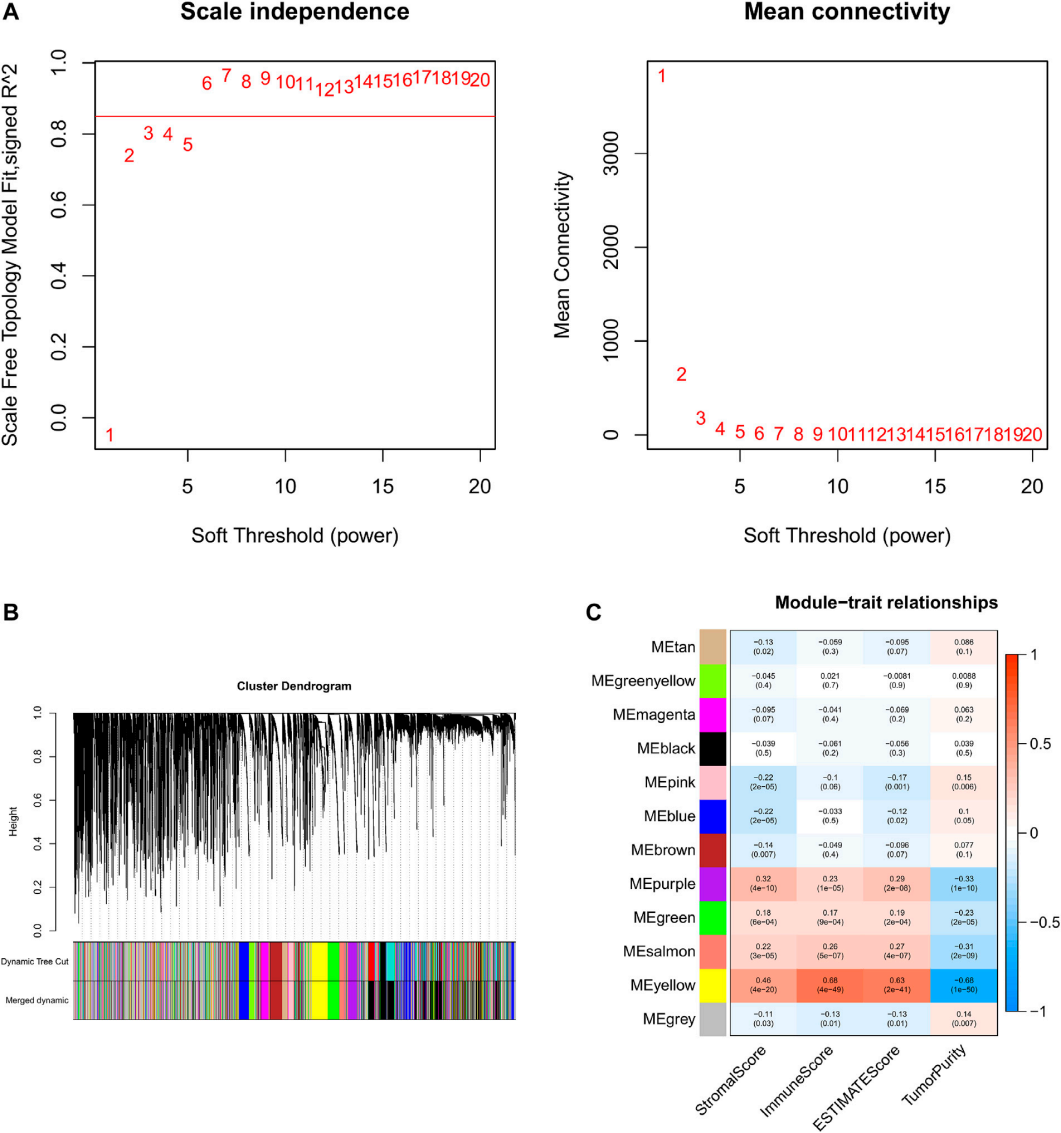
WGCNA and identification of the TME score-related module. **(A)** Analyses of the appropriate soft threshold power and minimum mean connectivity to construct topological overlap matrix (TOM). **(B)** Clustering dendrograms of the co-expression network modules. **(C)**: The correlation analysis between the TME score and module, correlation coefficient, and *p*-values are shown.

Based on the aforementioned analysis, the weighted gene co-expression network analysis (WGCNA) was performed to evaluate the gene expression matrix of 374 HCC samples. First, all the samples are hierarchically clustered by using the group-average method, and according to the clustering results, 45,000 is selected as the truncation value of the height of the clustering tree of the sample to exclude the samples that are obviously outliers (Supplementary Figure S2A). To construct a scale-free network, the soft threshold is calculated according to the pickSoftThreshold functions, and the minimum integer whose scale-free fitting coefficient R2 is above 0.8 is taken as the soft threshold. We choose the soft threshold power six to construct the adjacency matrix, where the scale-free topological fitting index is greater than 0.8 and the mean connectivity tends to the minimum (Figure 4A). Then, the adjacency matrix was converted into the topological overlap matrix (TOM) to minimize the impact of noise and false positives as much as possible. With topological isomerism matrix (TOM), we need to perform average-linkage hierarchical clustering and module identifications through a dynamic tree cut with a deepSplit parameter set as 2 and the minimum number of genes was set at 350 per module (Supplementary Figure S2B). Similar modules were merged following a height cutoff of 0.3 (Supplementary Figure S2C) and clustering dendrograms were presented (Figure 4B). To identify the TME-related modules, the module−trait relationships plot was presented by the Pearson correlation test (Figure 4C). Results suggested that the yellow module was most associated with the tumor microenvironment, with 4,030 genes in total.

### Identification of TME Score Related DEGs

**FIGURE 5:**
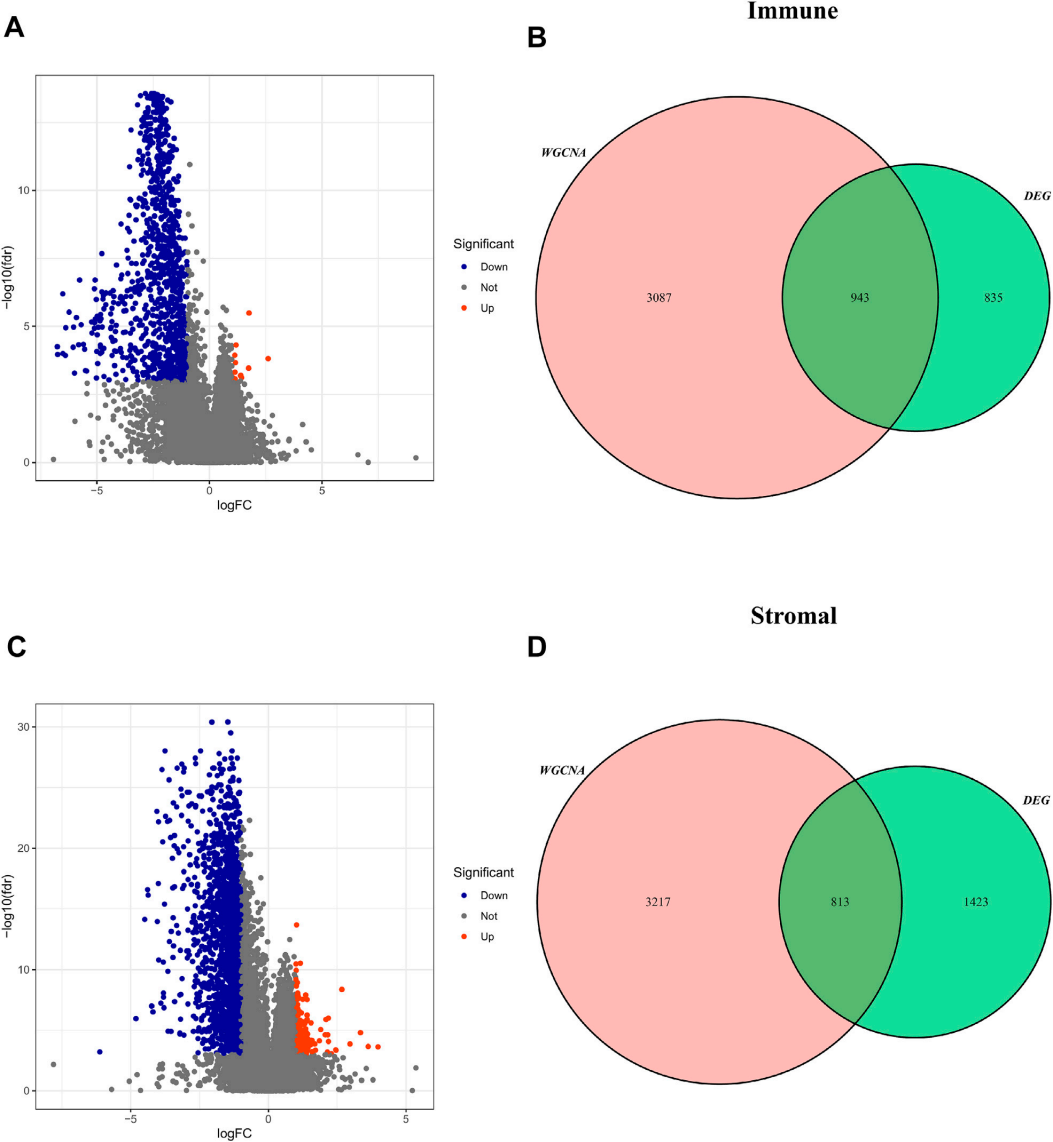
Screening for intersected DEGs. **(A,C)** The volcano plot of DEGs in high and low immune/stromal score groups. **(B,D)**: The intersected genes of immune/stromal score-related DEGs and WGCNA.

According to the optimal cut-off value, the TME score and tumor purity could be divided into high- and low-groups, and the differential genes between the two groups were screened (FDR <0.05, log |FC| < 1) (Supplementary Informations S1–S4). These TME-related DEGs were visualized *via* the volcano plots, in which red dots represent upregulated genes and blue dots represent downregulated genes (Figures 5A,C). Then, the intersected genes of immune/stromal score-related DEGs and WGCNA were obtained for subsequent analysis (Figures 5B,D).

### Development and Validation of the TME-Related Prognostic Signature for HCC

**FIGURE 6:**
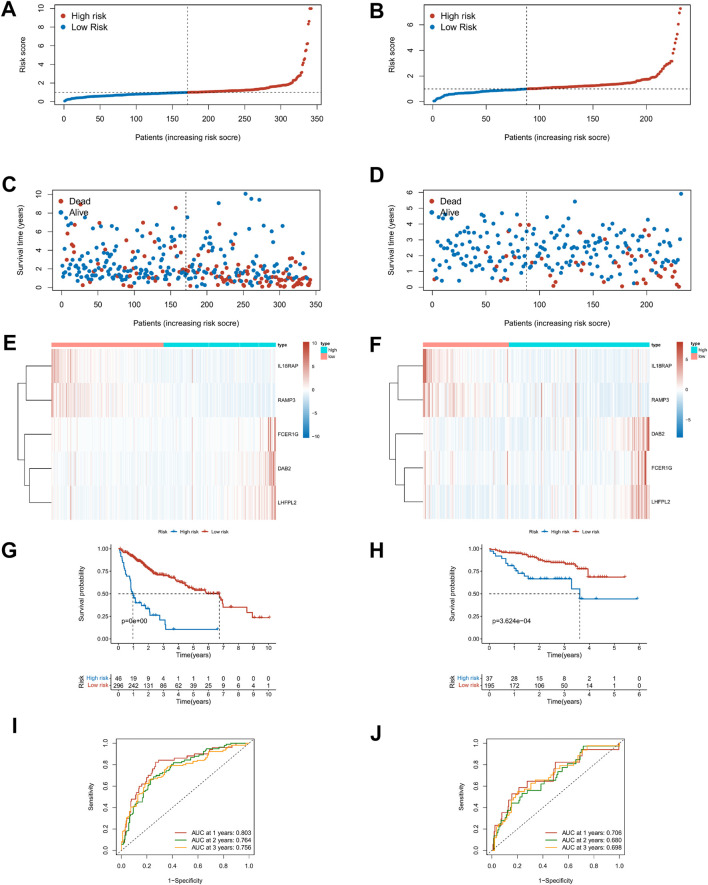
Prediction performances of risk signature in the training (TCGA, left) and validation cohorts (ICGC, right). **(A–D)** Distribution of risk scores and survival status in patients with HCC. **(E,F)** Heat maps of five-gene expression profiles in the high- and low-risk groups. **(G,H)** The K–M survival curves analysis in high‐ and low‐risk groups. **(I,J)** The time-dependent ROC curve analysis of TME risk signature for predicting 1-, 3-, and 5-year survival sensitivity.

The Lasso regression analysis was performed for DEGs associated with immune score and stromal score, respectively (Supplementary Figures S3A–D). For the immunity score, *DAB2*, *IL18RAP*, *KLRB1*, *P2RY6*, *RAMP3*, *FCER1G*, and *LHFPL2* were screened out, and *LINC001150*, *KLRB1, RAMP3*, and *LINC01094* were filtered out for the stromal score. To further explore the independent prognostic value of these nine genes, the univariate regression analysis was performed and 95% confidence intervals were calculated. The forest plot results showed that six of the genes were risk factors (HR > 1) and three were protective factors for HCC patients (HR < 1) (Supplementary Figure S3E). Then the model optimization was performed by incorporating forward to backward and getting the least AIC value according to different fitting results, and finally, the prognostic risk model related to tumor microenvironment constructed by 5 genes was obtained. Risk score = (0.257 × *DAB2*) + (−0.607 × *IL18RAP*) + (−0.314 × *RAMP3*) + (0.168 × *FCER1G*) + (0.327 × *LHFPL2*). The risk score of each HCC patient was calculated according to this formula. Figures 6A–D described the risk scores and survival status of HCC patients in the training (TCGA) and validation cohorts (ICGC), respectively. The heatmap findings in Figures 6E,F show that as risk factors, the expression of *DAB2*, *FCER1G*, and *LHFPL2* gains with a rising risk score, whereas *IL18RAP* and *RAMP3* decreased with the increase of risk score as a protective factor. Based on the optimal cut-off point, the Kaplan–Meier survival analysis illustrated that patients with low risk showed a higher survival possibility than those with high risk (TCGA training cohorts: *p* < 0.001; ICGC validation cohorts: *p* < 0.001, Figures 6G,H), suggesting that the TME risk signature had prognostic significance. According to the median value, the K–M survival analysis showed the same trend (Supplementary Figures 3F,G). Furthermore, we assessed the TME-risk signature’s prediction sensitivity and specificity using a time-based receiver operating characteristic (ROC) curve. For the training and validation sets, the AUC values for risk signatures at 1, 3, and 5 years were 0.803, 0.764, and 0.756 and 0.706, 0.680, and 0.698, respectively (Figures 6I,J). As a result, the potential of the TME risk signatures to forecast the prognosis of HCC was demonstrated.

### Generation of the Prognostic Nomogram

**FIGURE 7:**
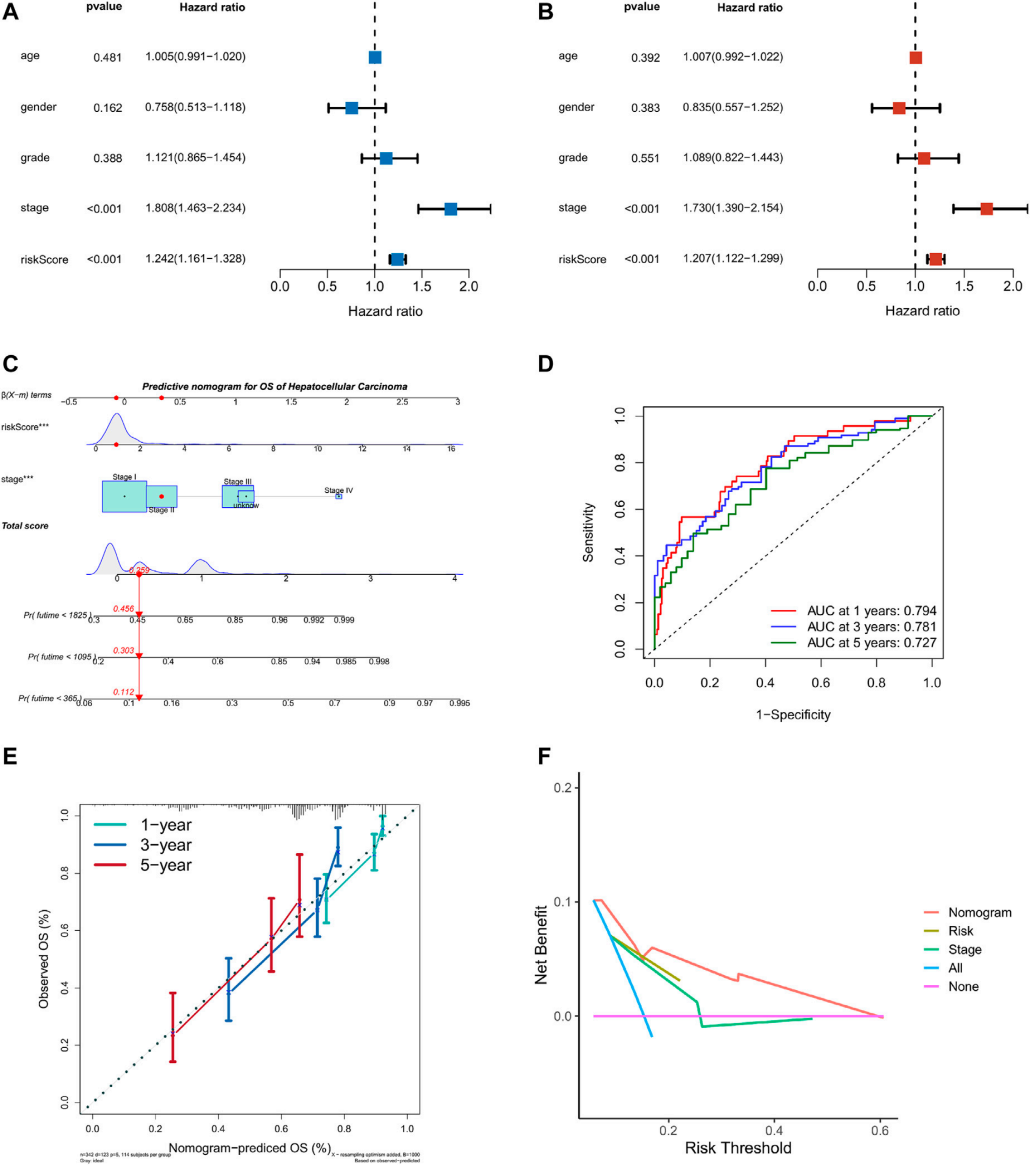
Nomogram of TME risk signature to predict HCC patients’ survival. **(A,B)** Forest maps of the univariate and multivariate Cox regression analysis, including risk score, age, gender, grade, and stage. **(C)** A nomogram, consisting of risk score and stage for predicting 1-, 3-, and 5-year survival for HCC patients. **(D)** The time-dependent ROC curve analysis indicated that the nomogram was a reliable and stable predictor for OS at 1-, 3-, and 5-years. **(E)** The calibration curve showed the nomogram’s predicted (*x*-axis) and actual survival probabilities (*y*-axis). **(F)** The DCA analysis evaluating the clinical utility of the nomogram.

Taking into account the prognostic significance of the TME-risk signature, we managed to combine risk scores with common clinical data to better predict the survival of HCC patients. We first performed univariate and multifactorial Cox regression analyses to analyze the effect of risk score and four clinical factors: age, gender, grade, and stage on prognosis (Figures 7A,B). Then, we constructed a nomogram based on risk score and stage to investigate the survival probability of 1-, 3-, and 5-year survivors. When the total points were 0.259, the corresponding 1-year, 3-year, and 5-year survival probabilities were 0.456, 0.303, and 0.112, respectively, as shown in Figure 7C. The area values of 1, 3, and 5-year survival rates under the ROC curve were 0.794, 0.781, and 0.727, respectively, indicating the accurate discernment (Figure 7D). Meanwhile, a calibration curve was drawn to evaluate the consistency of the OS predicted value and true value (Figure 7E). The DCA curve we plotted showed clinical benefits for patients with HCC (Figure 7F).

### Gene Set Enrichment Analyses

**FIGURE 8:**
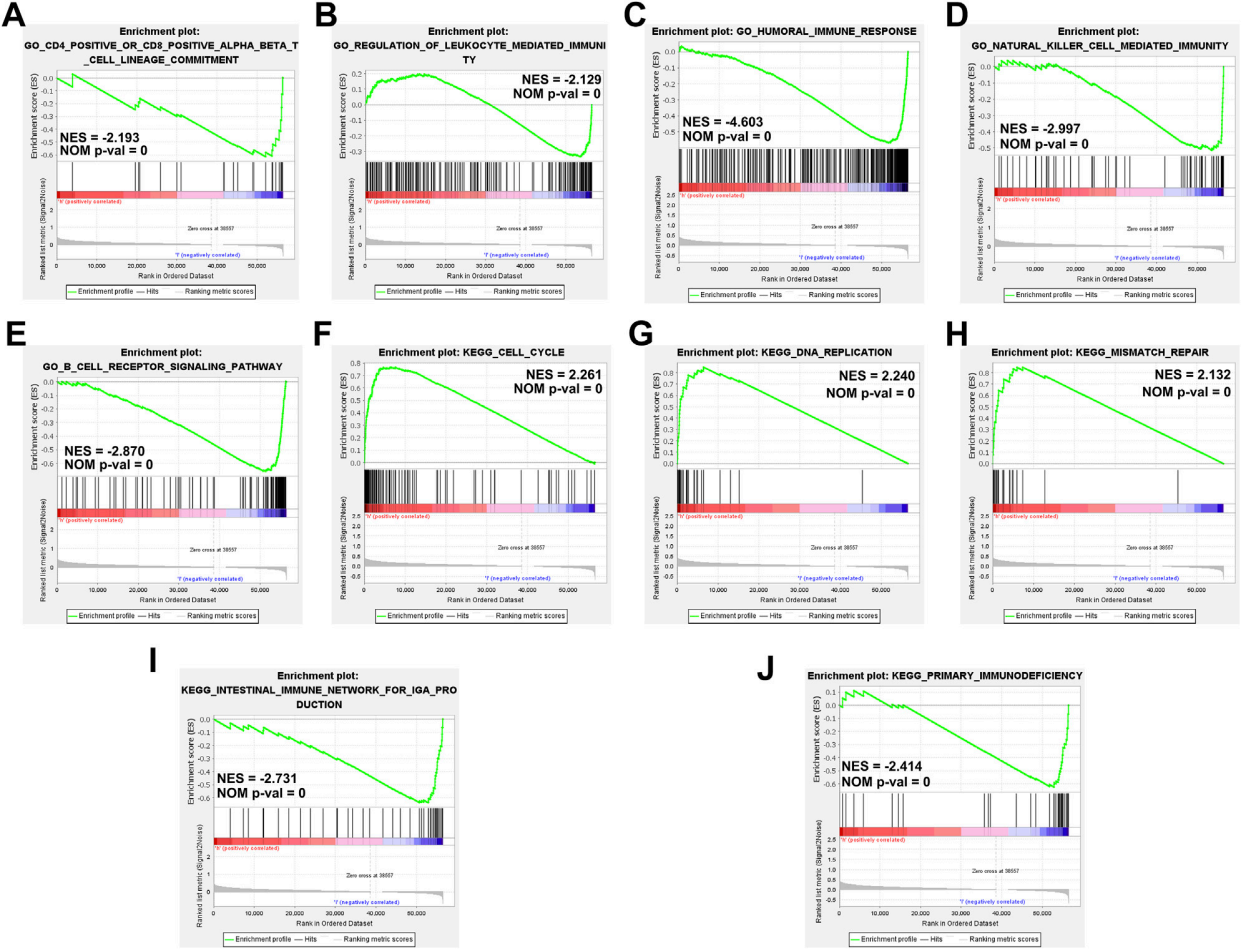
Gene set enrichment analyses of immune-related pathways based on high and low risk. **(A–E)** The GO enrichment analysis using the gene set “c5.go.v7.4. symbols.” **(F–J)** The KEGG pathway enrichment analysis using the gene set “c2.cp.kegg.v7.4. symbols.”

We performed GSEA based on the TME score risk signature to better understand the possible mechanism in patients with HCC. The GO terms were enriched mainly in the immunomodulatory-related pathways such as the CD4^+^/CD8^+^ αβT cell lineage commitment pathway (NES = −2.193, NOM *p*-val = 0), leukocyte-mediated immunity pathway (NES = −2.129, NOM *p*-val = 0), humoral immune response pathway (NES = −4.603, NOM *p*-val = 0), natural killer cell-mediated immunity signaling pathway (NES = −2.997, NOM *p*-val = 0), and B-cell receptor signaling pathway (NES = −2.870, NOM *p*-val = 0) (Figures 8A–E). The KEGG pathway enrichment analysis revealed that the prognostic signature was significantly involved in the cell cycle- (NES = 2.261, NOM *p*-val = 0), DNA replication- (NES = 2.240, NOM *p*-val = 0), mismatch repair- (NES = 2.132, NOM *p*-val = 0), intestinal immune- (NES = −2.731, NOM *p*-val = 0), primary immunodefiency (NES = −2.414, NOM *p*-val = 0)-related signaling pathways (Figures 8F–J). These results our signature play an essential role in the tumor immune microenvironment (Supplementary Information S5, S6).

### Risk Scores Correlated With Tumor Microenvironment, ICI Treatment Response, Single-Nucleotide Polymorphisms, and Drug Sensitivity

**FIGURE 9:**
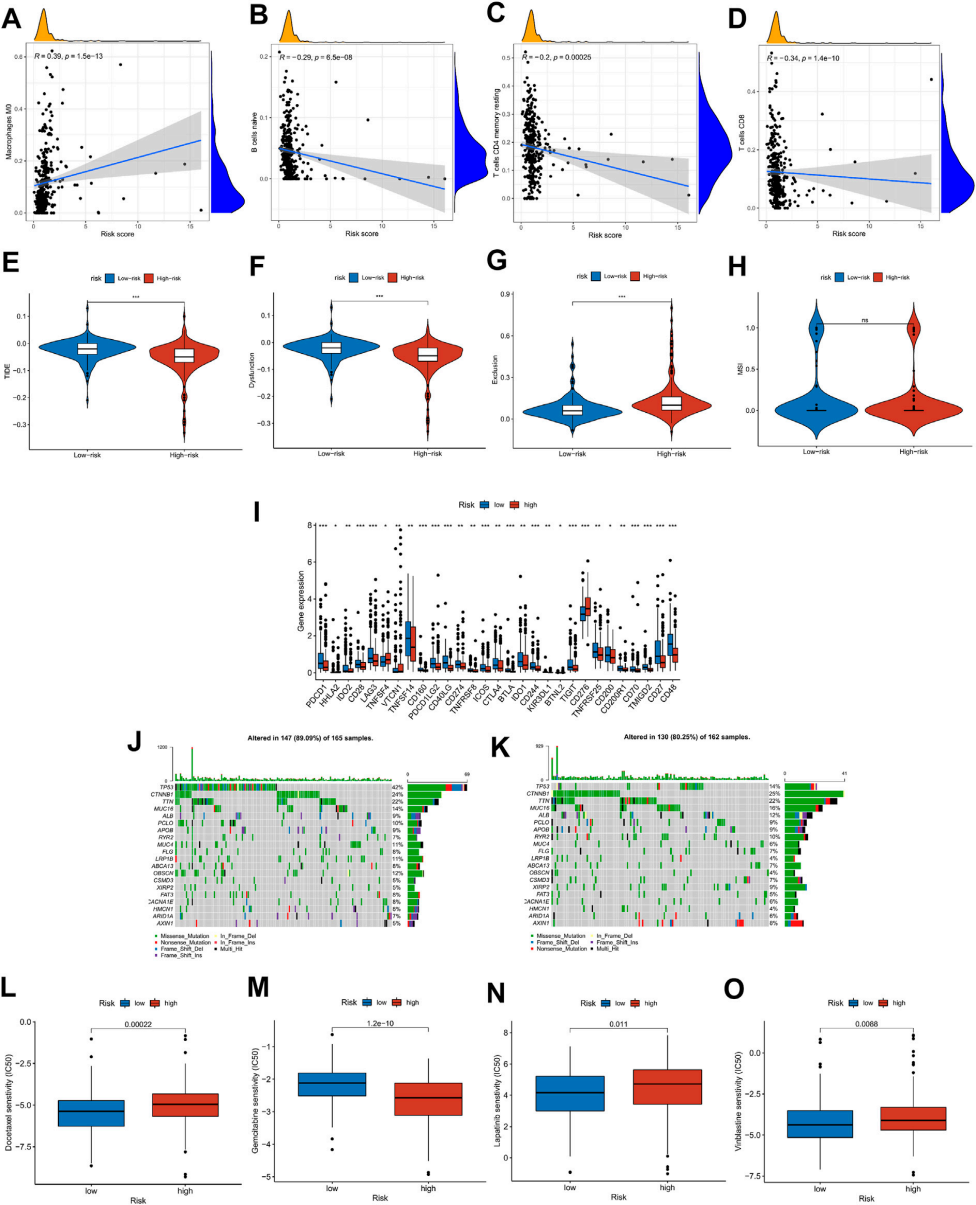
Risk score with immune infiltration, immunotherapy, SNP, and drug sensitivity. **(A–D)** Relationship between risk score and TME immune cell infiltration. **(E–H)** TIDE score distribution in the high- and low-risk groups. **(I)** The expression level of possible immune checkpoints in high- and low-risk groups. **(J–K)** Waterfall maps of twenty mutated genes in high-(left)/low-risk (right) groups. **(L–O)** Sensitivity of chemotherapy and targeted therapy in high- and low-risk groups.

To explore the relationship between risk score and tumor microenvironment, we first used the R package “immunedeconv” and “ggplot2” to draw the heat map of immune infiltration of patients in the high- and low-risk groups (Supplementary Figure S4A). At the same time, CIBERSORT was utilized to calculate the proportion of 22 types of immune cells in each HCC patient (Supplementary Figure S4B). In Figures 9A–D, Macrophages M0 was positively correlated with risk score, while B-cell naïve, T-cell CD4 memory resting, and T-cell CD8 were on the contrary (|R| ≥ 0.02, *p* < 0.001), which was consistent with the boxplot in Supplementary Figure S4C. The ICI treatment response was assessed by the tumor immune dysfunction and exclusion (TIDE) score between the high- and low-risk groups. Results indicated that TIDE and Dysfunction were exceedingly expressed in the group with low risk, whereas Exclusion was highly expressed in the group with high risk (*p* < 0.05, Figures 9E–H). Then, we explored the differential expression of immune checkpoints between high- and low-risk groups. In the low-risk group, PDCD1 (PD-1), CD274 (PD-L1), PDCD1LG2 (PD-L2), and CTLA4 were highly expressed, suggesting that low-risk patients may respond better to immunotherapy (Figure 9I). These findings suggest that patients at low risk may respond better to immunotherapy. Moreover, the condition of SNPs was also investigated (Figures 9J,K). Among the 165 patients with high risk, 147 (89.09%) had the gene mutation, and the mutation frequency of TP53 (42%) was significantly higher than that of the low-risk group (14%). Finally, the relationship between risk score and clinical drug sensitivity was analyzed. As shown in Figures 9L–O, Docetaxel, Lapatinib, and Vinblastine sensitivity were positively associated with the risk score, whereas Gemcitabine was highly sensitive in the low-risk cluster.

### Gene Expression in TME Risk Signature

**FIGURE 10:**
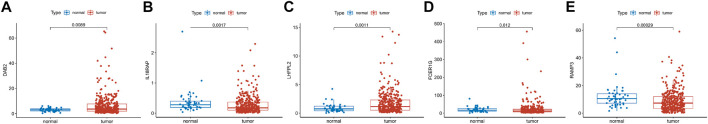
Gene expression in risk models in TCGA database. **(A–E)** Expression levels of DAB2, IL18RAP, LHFPL2, FCER1G and RAM3 in tumor tissues and normal tissues in TCGA database.

We explored the expression of five genes in the constitutive risk model in high and low-risk groups. As shown in Figures 10A–E, *IL18RAP*, *FCER1G*, and *RAMP3* were upregulated in the low-risk group, whereas *DAB2* and *LHFPL2* were highly expressed in the high-risk group. We also verified the expression levels of these genes in normal liver cells (LO2) and liver cancer cells (MHCC-97h, HLF, and Huh7) (Supplementary Figures S5A–E). Primer sequences of these genes are shown in Supplementary Table S1.

## Discussion

Hepatocellular carcinoma is a highly heterogeneous disease whose pathophysiological mechanism is still largely unknown (Llovet et al., 2016; Forner et al., 2018; Llovet et al., 2021). In the past decades, breakthroughs have been made in targeted therapy and immunotherapy, but the prognosis of HCC patients is still poor and varies greatly (Zhang et al., 2020; Zongyi and Xiaowu, 2020). Therefore, in addition to TNM classification, the development of diverse risk models to forecast the prognosis of HCC patients is of great importance for personalized treatment and follow-up. More and more evidence suggests that the tumor microenvironment is associated with proliferation, angiogenesis, invasiveness, metastasis, drug resistance, and immune escape. TME consists of immune cells, stromal cells, endothelial cells, blood vessels, soluble molecules, etc. Among them, immune cells and stromal cells play an important role in the occurrence and development of HCC patients (Whiteside, 2018; Lu et al., 2019; Song et al., 2021). The ESTIMATE algorithm was established using gene expression data to estimate immune and stromal cells and generates immune and stromal scores to predict immune and stromal cell infiltration in the TME. In recent years, studies have used the ESTIMATE algorithm to probe the tumor microenvironment in breast, gastric, and colorectal cancers; however, immune/stromal infiltration assessment in HCC is far from adequate. In this study, we first downloaded the TCGA RNAseq and clinical data, calculated the score of each patient through the ESTIMATE algorithm, and explored its correlation with clinical characteristics. Second, WGCNA was used to find the modules most relevant to TME score. The intersected genes of immune/stromal score and WGCNA were obtained for subsequent analysis. The Lasso regression analysis was performed on the intersection genes, and finally, a prognostic risk model of TME score related was obtained. We evaluated the sensitivity and specificity of prognostic model predictions and validated those using external data ICGC. In our signature, five genes were identified, including *IL18RAP*, *FCER1G*, *RAMP3*, *DAB2*, and *LHFPL2*. Related studies have shown that interleukin 18 receptor accessory protein (IL18RAP) encodes an accessory subunit of the interleukin-18 (IL18) receptor, which can enhance the IL18 binding activity of IL18 receptor and play a role in IL18 signal transduction (Lin et al., 2020). Receptor activity modifying protein 3 (*RAMP3*) is a type I transmembrane protein that can transport calcitonin receptor-like receptor (CRLR) to the plasma membrane. The regulatory role of RAMP3 is significantly different in different cancers. In Aiping Fang’s research, *RAMP3* was associated with the overall survival (OS) and relapse-free survival (RFS) of HCC patients (Fang et al., 2018). LHFPL tetraspan subfamily member 2 (*LHFPL2*) is a member of the lipoma HMGIC fusion partner (LHFP) gene family. It is associated with macrophages in triple-negative breast cancer and chronic proliferation in acute myeloid leukemia (Hatfield et al., 2014). DAB adaptor protein 2 (*DAB2*, *DOC2*, and *DOC-2*) encodes a phosphorylated protein of the mitogenic response and participates in many signaling pathways (Ogbu et al., 2021). Numerous studies have shown that *DAB2* acts as an oncogenic factor to inhibit tumor cell proliferation in the early tumor stage, but in the late tumor stage, *DAB2* promotes tumor cell EMT and invasion leading to metastasis. In HCC, *DAB2*, as a tumor suppressor gene, is associated with the activation of the Ras signaling pathway, and mir-106b can promote the proliferation and migration of HCC cells by targeting *DAB2* (Calvisi et al., 2007). Fc epsilon receptor Ig (*FCER1G* and *FCRG*) is a key molecule involved in the progression and immune response of various tumors (Xu et al., 2021b). Dong et al. (2022) reported that *FCER1G* is positively associated with macrophage infiltration and contributes to poor prognosis by modulating tumor immunity in clear cell renal cell carcinoma. Through our PCR verification results, it can be seen that the expressions of *DAB2* and *FCER1G* are not completely consistent with the TCGA database, which may be related to cell heterogeneity.

More efforts are needed to explore the mechanism of these genes in the immune microenvironment of HCC patients. Furthermore, we explored the possible mechanisms of risk score and its correlation with immune cells, immune efficacy, SNP, and drug sensitivity. In conclusion, we constructed a hepatocellular carcinoma TME score-related prognostic model. It may provide potential therapeutic targets and prediction of immune efficacy. Although an excellent prognostic model has been established, there are still some limitations in our study. First, we are only based on data from the TCGA and ICGA databases, lacking multi-center verification. Second, we only verified the expression of genes in the model at the mRNA level and did not explore the mechanism in depth. In the future, we will expand the sample size to verify and optimize our model, and further, explore the mechanism of its occurrence and development. Despite these limitations, our study provides important clues to clarify the relevant molecular mechanisms in HCC and helps to develop new treatment strategies.

## Data Availability

The datasets presented in this study can be found in online repositories. The names of the repository/repositories and accession number(s) can be found in the article/Supplementary Material.
