# Supplementary material for: Identification and Validation of a Novel Tumor Microenvironment-Related Prognostic Signature of Patients With Hepatocellular Carcinoma
Source: Front Mol Biosci. 2022 Jun 30;9:917839. doi: 10.3389/fmolb.2022.917839 (PMC9280086; doi:10.3389/fmolb.2022.917839)
Supplement: Supplementary file 1 [file DataSheet1.ZIP › supplementary Table 1.docx]

Table 2 : Primer sequences

| **Name** | **Primer sequences (5'to3')** |
| --- | --- |
| *DAB2*-F | CTCTGTCCAGTCCTCACCACAT |
| *DAB2*-R | GTTCTGAGACGGGAGGAGCAAA |
| *FCER1G*-F | GTGCGAAAGGCAGCTATAACCAG |
| *FCER1G*-R | GGTGGTTTCTCATGCTTCAGAGT |
| *RAMP3*-F | ACGTCTGGAAGTGGTGCAACCT |
| *RAMP3*-R | CCAGTAGCAGCCCACGACATTG |
| *LHFPL2*-F | GCATCTTCAATGTCTGTGGGCTG |
| *LHFPL2*-R | GTCCACAGTAGTCTATGGCCTTC |
| *IL18RAP*-*F*  *IL18RAP*-*R*  GAPDH-F | GCACAAAGTCCAGCGGTAACCT  GTCCACGAACTCACAGTATCCG  ATAGCACAGCCTGGATAGCAACGTAC |
| GAPDH-R | CACCTTCTACAATGAGCTGCGTGTG |
