# Supplementary figures and images for: Identification and Validation of a Novel Tumor Microenvironment-Related Prognostic Signature of Patients With Hepatocellular Carcinoma
Source: Front Mol Biosci. 2022 Jun 30;9:917839. doi: 10.3389/fmolb.2022.917839 (PMC9280086; doi:10.3389/fmolb.2022.917839)

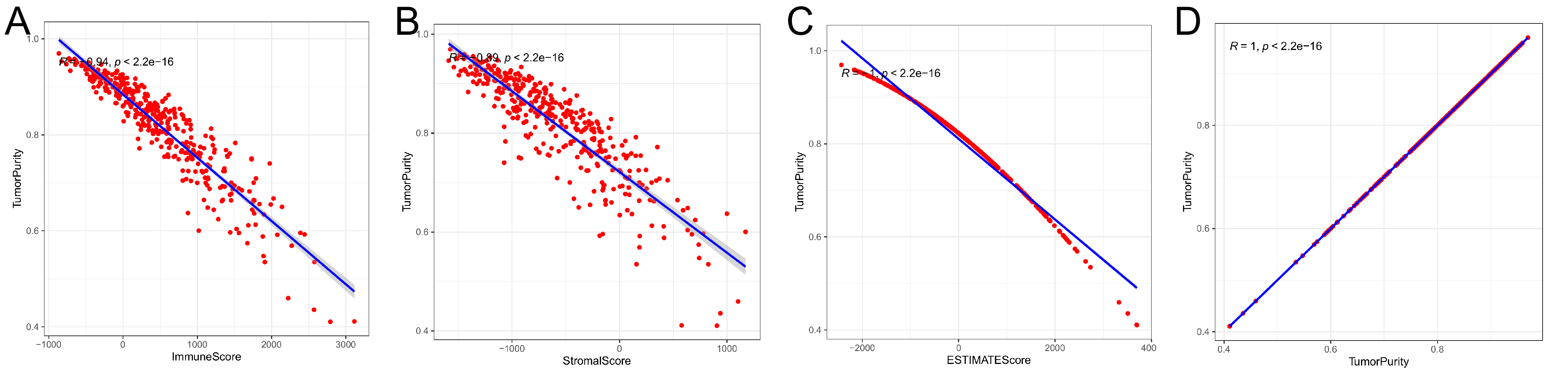

Supplement: Supplementary file 1 [file DataSheet1.ZIP › supplementary Figure 1.tiff]

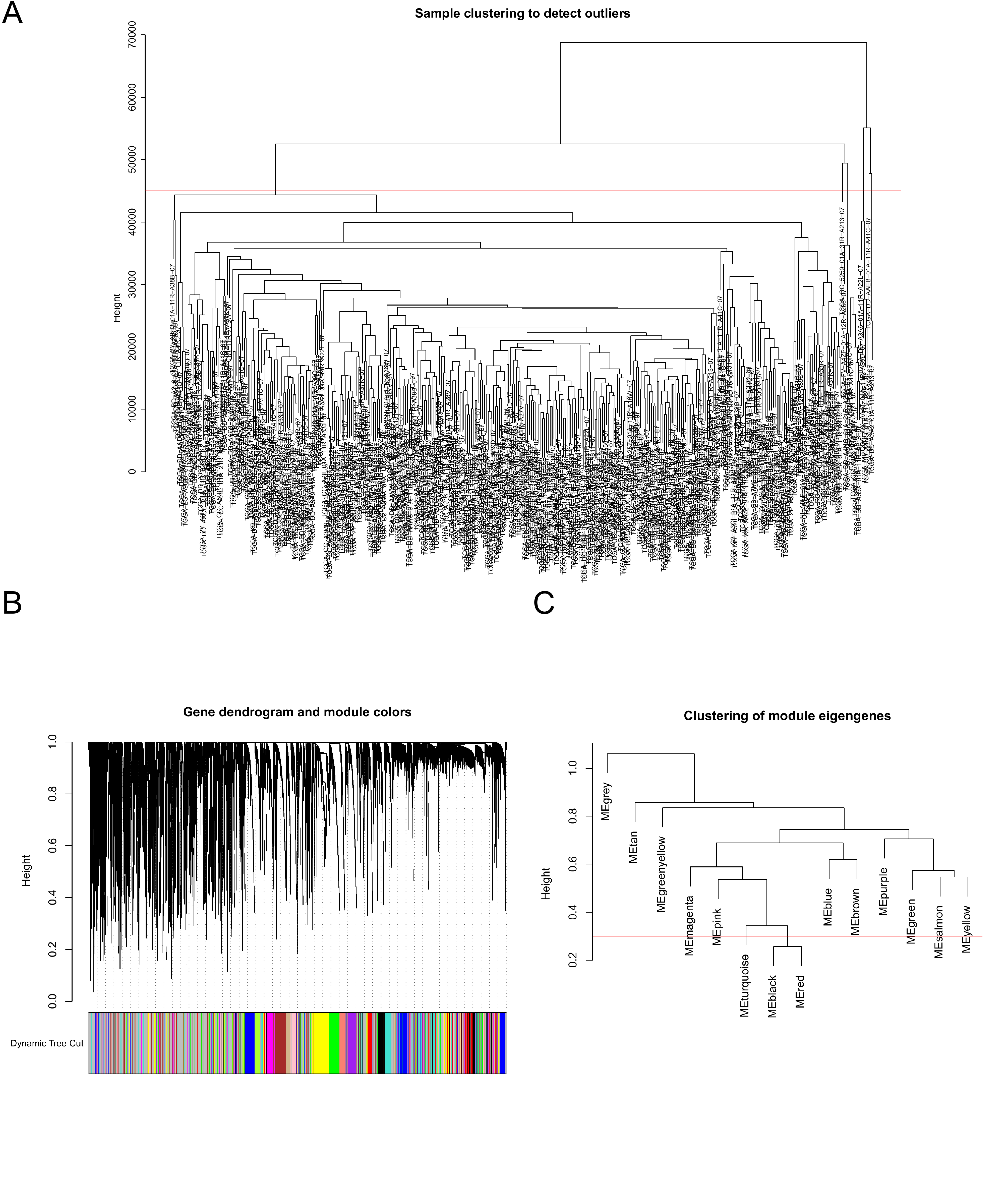

Supplement: Supplementary file 1 [file DataSheet1.ZIP › supplementary Figure 2.tiff]

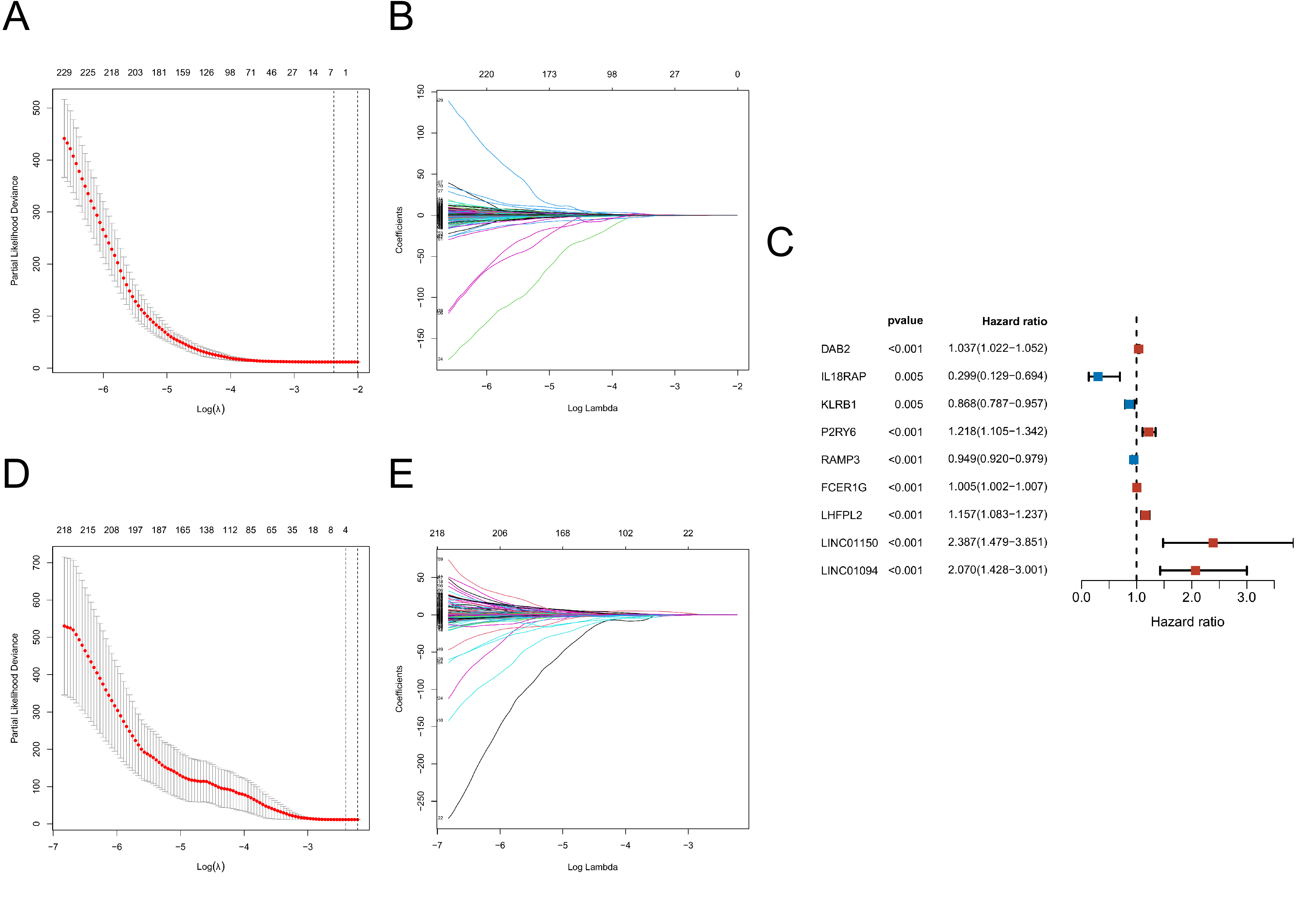

Supplement: Supplementary file 1 [file DataSheet1.ZIP › supplementary Figure 3.tiff]

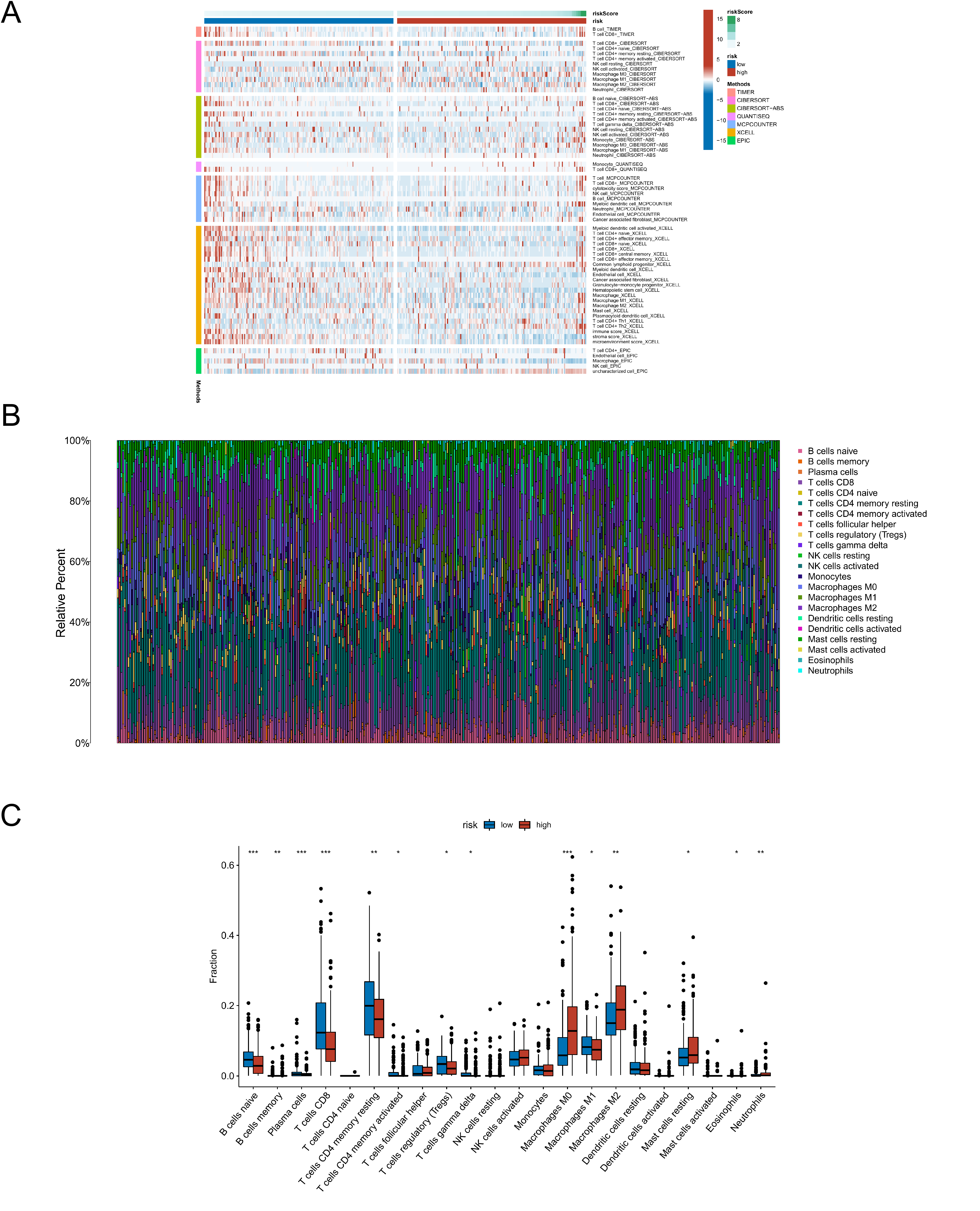

Supplement: Supplementary file 1 [file DataSheet1.ZIP › supplementary Figure 4.tiff]

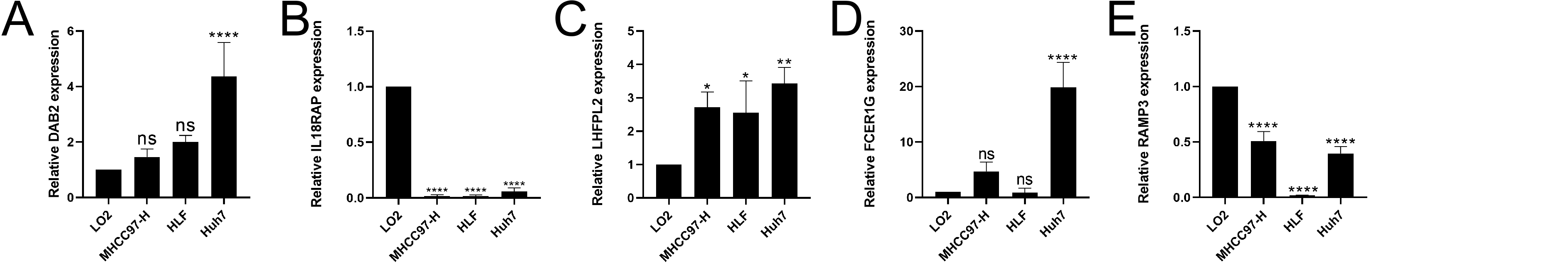

Supplement: Supplementary file 2 [file Image2.TIF]

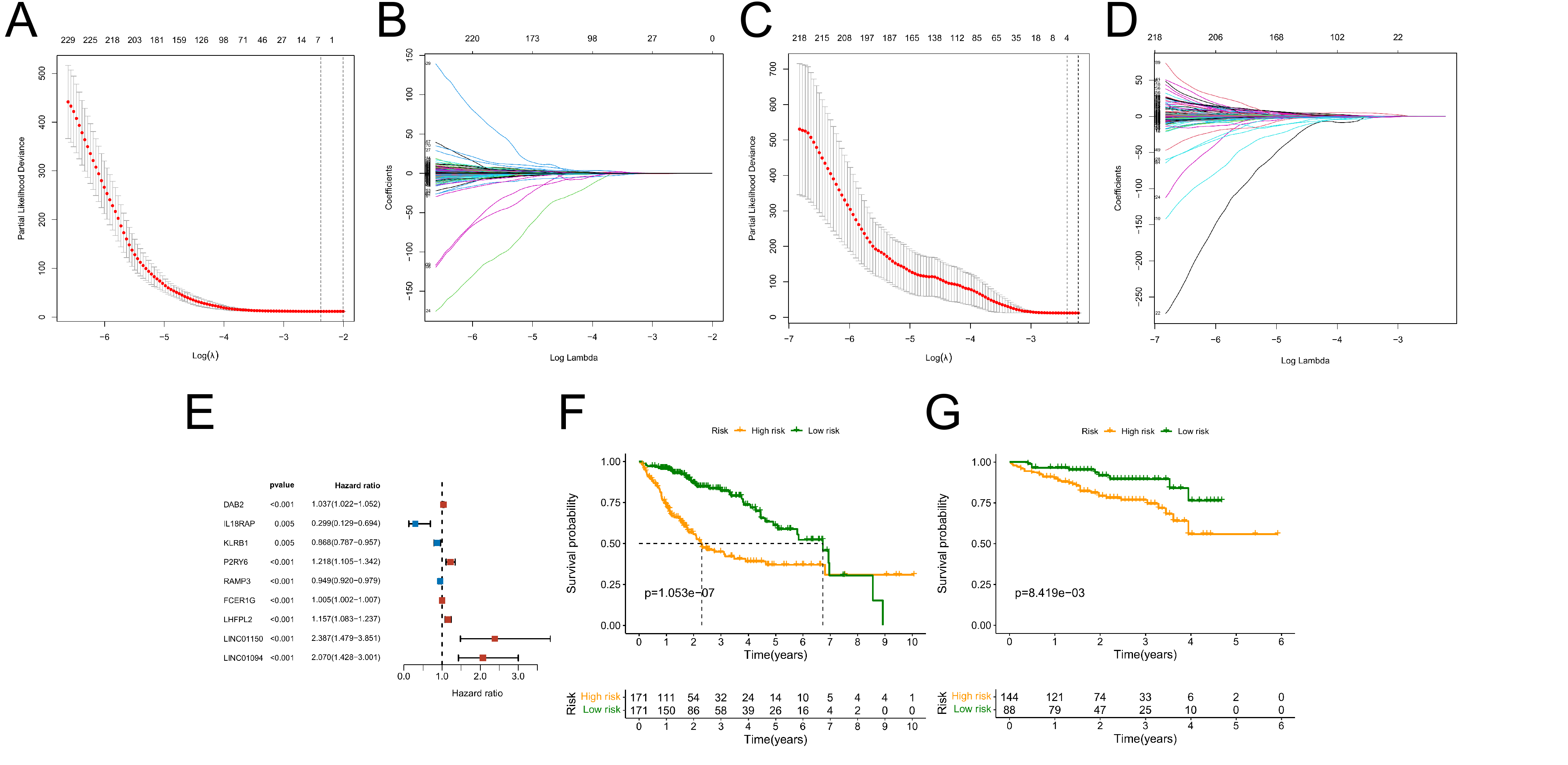

Supplement: Supplementary file 3 [file Image1.TIF]
